# Primary hyperoxaluria detected by bone marrow biopsy: case report

**DOI:** 10.1186/s12907-017-0059-7

**Published:** 2017-09-20

**Authors:** F. Nachite, M. Dref, A. Fakhri, H. Rais

**Affiliations:** Department of Pathology, FMFM-UCAM-CHU Mohammed VI-50 Assif, 40000 Marrakech, Morocco

**Keywords:** Primary hyperoxaluria, Bone marrow biopsy, Oxalate crystals

## Abstract

**Background:**

Primary hyperoxaluria is a rare disease with an estimated prevalence of 1 to 3 cases per million. It is due to a hepatic enzyme deficiency responsible for an endogenous overproduction of oxalate. Oxalate crystals commonly deposit in the kidney and more rarely in bone marrow.

The literature has reported, to the best of our knowledge, only two cases of hyperoxaluria diagnosed by bone marrow biopsy and our case is the only one that does not show radiological bone lesions.

**Case presentation:**

A young 22 year old chronic hemodialysis patient with nephrocalcinosis. The patient had a personal and family history of recurrent kidney stones. He presented bone pain with worsening of his general state. On physical examination, no organomegaly was detected. Biological check-up showed only a normochromic and normocytic regenerative anemia resistant to treatment and a bone marrow biopsy was performed. It showed deposits of crystals of oxalate in the bone marrow surrounded by inflammatory reaction against foreign bodies. Given our context, no liver biopsy or genetic studies, which are gold standard of diagnosis testing, were done. The diagnosis of primary hyperoxaluria was made based on morphological characteristics of crystals, his medical and family history, and the absence of any secondary cause of the condition. Since curative treatment is not available in our country, the patient only receives a palliative treatment.

**Conclusion:**

Primary hyperoxaluria is rarely evoked by the histological study of a bone marrow biopsy. The lack of the possibility of the only effective treatment in our context and the diagnosis, usually late, of this pathology are at the origin of the fatal evolution of the disease in almost all the cases.

## Background

Primary hyperoxaluria is a rare autosomal recessive metabolic disorder [1]. The estimated prevalence of 1 to 3 cases per million. It is an inherited error of metabolism due to defective liver enzyme activity [1]. The consequence of this disease is an endogenous overproduction of oxalate, as opposed to secondary hyperoxaluria, which is due to excessive dietary intake or increased intestinal absorption of oxalate [1]. The highly insoluble oxalate crystals are deposed in various organs. The bone marrow localization of these deposits remains rare [2]. The diffuse deposition of oxalate crystals in the bone marrow, surrounded by giant-cell granulomas and bone resorption pits in bone trabecula is the cause of radiologic changes in this disease [2]. The radiological signs pathognomonic of oxalosis are a dense metaphyseal bands, lucent metaphyseal bands and vertebral osteocondensations [3]. Diagnosis is often made at a late stage of disease with serious adverse consequences.

The interest of our case is to report the rarity of the diagnosis of primary hyperoxaluria in an anuric individual who did not show the bone lesions in the radiographies previously described for such a case.

The literature has reported, to the best of our knowledge, only two cases of hyperoxaluria diagnosed by bone marrow and our case is the only one that does not show bone lesions.

## Case presentation

Clinical history: A 22 year-old white man, born from a second degree consanguineous marriage and with a family history of kidney stones. The patient had recurrent kidney stones since the age of 6, progressing towards chronic renal failure. He is a chronic hemodialysis for 4 years with nephrocalcinosis. He was hospitalized for invalid bone pain, fatigue, and pallor progressing for 4 months. Physical examination shows no organomegaly and no adenopathy.

Biological, radiologic and histopathologic findings:

Complete blood count showed a normochromic and normocytic regenerative anemia with hemoglobin level of 7.6 g/dL. Remarkable serum biochemistry lab data included ferritin level of 1730 ng/mL. The phosphocalcic balance was normal. Given the patient’s history, the symptomatology and the anemia, two diagnoses were suggested: malignant hemopathy or primary hyperoxaluria. Urinary sediments examination in search of oxalate crystals were not performed due to anuria of our patient. The standard radiology of the hands, wrists and spine do not show any abnormality. An osteomedullary biopsy was performed in search of malignant hemopathy.

**Fig. 1:**
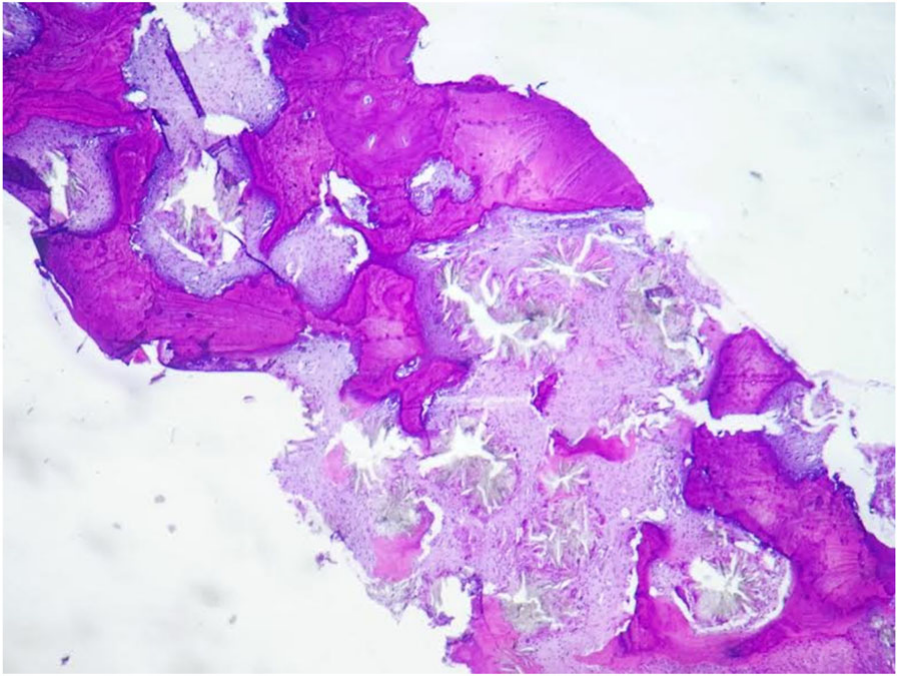
Bone chambers massively invaded by grayish-colored oxalate crystals deposited in stars or rosettes (hematoxylin and eosin stain, original magnification × 40)

**Fig. 2:**
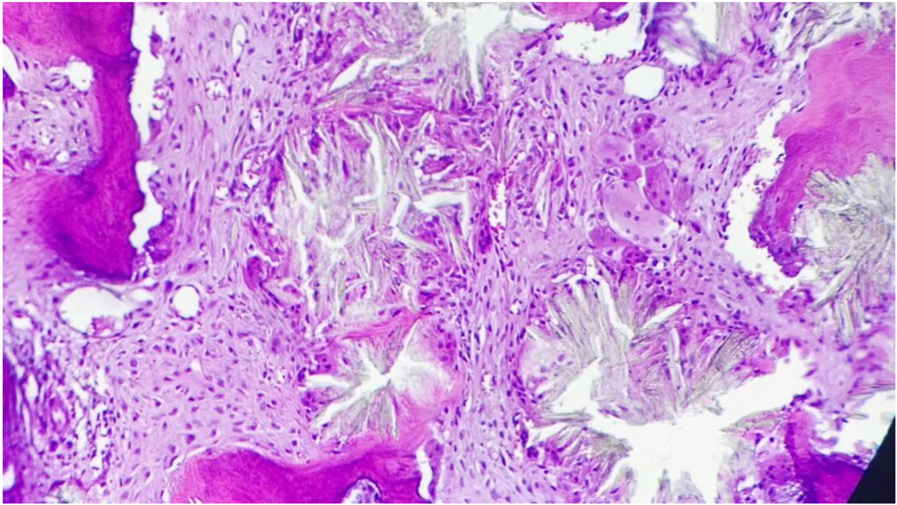
Grayish-colored oxalate crystals deposited in stars or rosettes surrounded by a macrophage reaction against foreign bodies (hematoxylin and eosin stain, original magnification × 200)

**Fig. 3:**
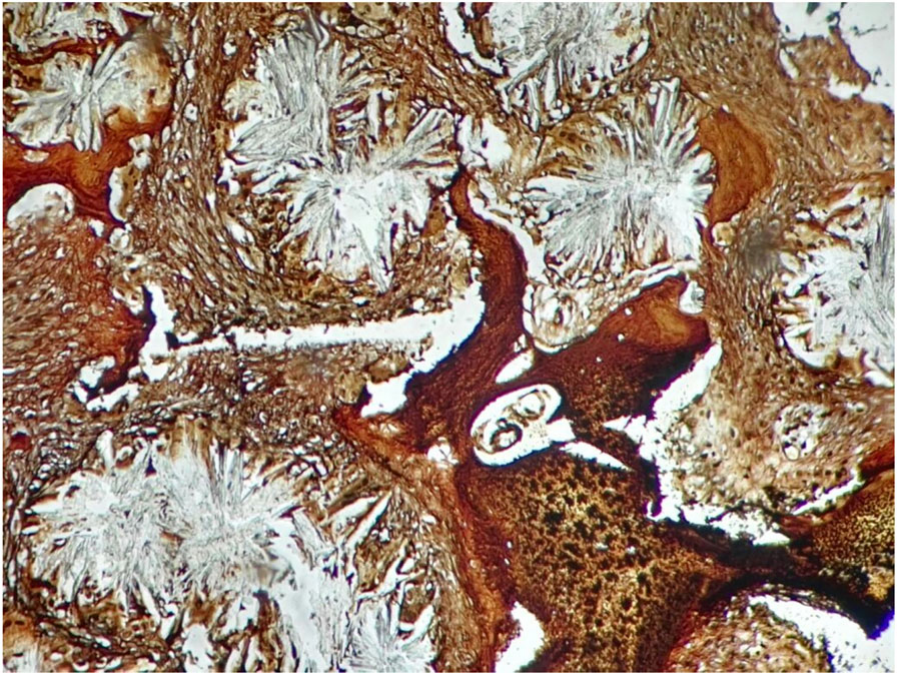
Medullar fibrosis (special trichrome stain, original magnification × 200)

**Fig. 4:**
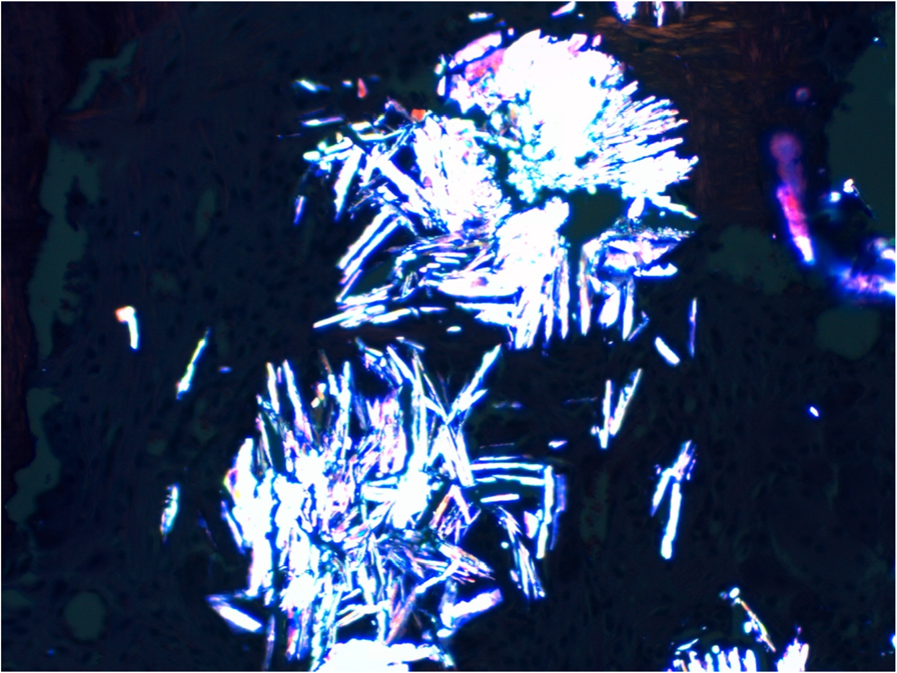
A very intense pale green birefringence of crystals of oxalate showed in polarized light (hematoxylin and eosin stain, original magnification × 400)

The bone marrow biopsy measure 0.7 cm in length. Hematoxylin and eosin stain shows six bone chambers, massively invaded by grayish-colored oxalate crystals deposited in stars or rosettes surrounded by a brisk foreign body giant cell reaction (Figs. 1 and 2). Special trichrome staining revealed a medullar fibrosis (Fig. 3). Under polarized light, crystals of oxalate showed a very intense pale green birefringence (Fig. 4).

Given our context, no liver biopsy or genetic studies, which are gold standard of diagnosis testing, were done. Only plasma oxalate level was made, he showed level of 320 μg/dL. The diagnosis of primary hyperoxaluria was made based on the characteristic morphology of crystals, plasma oxalate levels, his medical and family history and the absence of any secondary cause of the condition.

## Discussion

Primary hyperoxaluria is a rare disease with an estimated prevalence of 1 to 3 per one million people and an incidence rate of about 1 per 100,000 births [1]. It affects at least 1% of pediatric population with end-stage renal disease. Three forms have been described in the literature, each corresponding to a particular enzymatic deficiency, but all are due to autosomal recessive transmissions. The most common disorder is due to a deficiency of the enzyme alanine: glyoxylate aminotransferase (PH type I), which is specific to the liver peroxisome 1–4 [1].

Oxalate crystals deposition is reported in the kidneys, cartilages, soft tissues and more rarely in bone marrow [4].

The clinical symptomatology in our patient is similar to the data described in the literature. Clinical symptoms start at an average age of 5 years and signs are recurring lithiasis, nephrocalcinosis, hematuria and urinary tract infections. These clinical manifestations lead to renal failure in childhood or adolescence [2, 5, 6].

While plasma oxalate levels are likely to be more accurate when patients develop chronic kidney disease, the increased urinary oxalate levels help to make the diagnosis [4]. In our case serum oxalate was very high and the urinary sediment could not be performed given the anuria.

In the literature, the radiograph shows pathognomonic skeletal manifestations of this pathology: dense metaphyseal bands, vertebral osteocondensation or osteolysis [2, 3]. These bone lesions are due to the macrophage inflammatory reaction induced by deposition of oxalate crystals causing bone resorption [2, 3]. Computed tomography (CT) can be very useful in assessing the extent of calcifications and tissue oxalate deposits [4]. In the serial radiographs of our patient, no anomaly was detected.

Unfortunately, in the case of our young patient, the diagnosis was delayed. Disabling bone pain associated with severe deterioration of the general state and treatment-resistant anemia prompted a bone marrow biopsy to rule out a hemopathy. This biopsy showed the presence of oxalate crystals deposits at the bone marrow with fibrosis and without the presence of bone lesions in the radiological assessment and suggested the diagnostic.

Given the rarity of bone marrow localization of these crystals, only two cases of primary Hyperoxaluria diagnosed by a bone marrow biopsy were found in literature, these two cases showed bone lesions on the radiographies.

Definitive diagnosis of primary hyperoxaluria is made by genetic studies and if genetic studies prove inconclusive, liver biopsy is undertaken to establish diagnosis [6].

A genetic counseling was considered for this patient, but unfortunately in our condition, it takes two years.

Treatment must be early and several measures are proposed. Medical treatments including large volume fluid intake, vitamin D, prescription of Pyridoxine for converting glyoxylate to glycine, and regular dialysis to reduce serum and urine oxalate concentration [4].

In chronic kidney disease, the best treatment to date was achieved with combined liver-kidney transplantation [4].

Recent progress affords us a wider perspective on molecular or non-invasive gene therapies [7].

## Conclusion

In this investigation, the histological study of the bone marrow biopsy evoked the diagnosis. The impossibility, in our context, of the application of the only effective treatment as well as the late diagnosis of this pathology, are at the origin of the fatal evolution of almost all patients affected by this disease.

## Abbreviations

CT: Computed tomography
PH: Primary hyperoxaluria

## References

[1] Cochat P, Deloraine A, Rotily M (1995). Epidemiology of primary hyperoxaluria type. Nephrol. Dial. Transplant.

[2] Brancaccio D, Poggi A, Ciccarelli C, Bellini F (1981). Bone changes in end stage oxalosis. AJR Am J Roentgenol.

[3] El Hage S, Ghanem I, Baradhi A, Mourani C, Mallat S, Dagher F, Kharrat K (2008). Skeletal features of primary hyperoxaluria type 1, revisited. J Child Orthop.

[4] Cochat P, Hulton SA, Acquaviva C, Danpure CJ, Daudon M, De Marchi M (2012). Primary hyperoxaluria type 1: indications for screening and guidance for diagnosis and treatment. Nephrol Dial Transplant.

[5] Gargah T, Khelil N, Youssef G, Karoui W, Lakhoua MR, Abdelmoula J. Primary hyperoxaluria type 1 in Tunisian children. Saudi J Kidney Dis Transpl [serial online] 2012 [cited 2017 Feb 7]; 23: 385–390 Available from: http://www.sjkdt.org/text.asp?2012/23/2/385/93188. 22382246

[6] Bogle MA, Teller CF, Tschen JA, Smith CA, Wang A (2003). Primary hyperoxaluria in a 27-year old woman. J Am Acad Dermatol.

[7] Salido E, Rodriguez-Pena M, Santana A, Beattie SG, Petry H, Torres A (2011). Phenotypic correction of a mouse model for primary hyperoxaluria with adeno-associated virus gene transfer. Mol Ther.

